# Characterization of a whole blood assay for quantifying myeloid-derived suppressor cells

**DOI:** 10.1186/s40425-019-0674-1

**Published:** 2019-08-28

**Authors:** Minjun C. Apodaca, Amy E. Wright, Angela M. Riggins, William P. Harris, Raymond S. Yeung, Lei Yu, Chihiro Morishima

**Affiliations:** 1 Department of Laboratory Medicine, University of Washington, Box 357110, 1959 NE Pacific St, Seattle, WA 98195 USA; 2 Department of Medicine, Division of Medical Oncology, University of Washington, Seattle, WA USA; 3 Department of Surgery, University of Washington, Seattle, WA USA; 4 Department of Medicine, Division of Gastroenterology, University of Washington, Seattle, WA USA

**Keywords:** Myeloid-derived suppressor cells, Immunotherapy, Liver cancer, Flow cytometry, Whole blood

## Abstract

**Background:**

Myeloid-derived suppressor cells (MDSC) have been found to play an important role in limiting immune responses in cancer. Higher circulating MDSC levels have been associated with greater tumor burden, poorer response to immunotherapy, and poorer survival. Optimal measurement of MDSC levels could provide clinicians with a useful prognostic and/or management tool.

**Methods:**

A whole blood (WB) nine color, 11 parameter flow cytometric assay was designed, utilizing fluorescently-labeled antibodies against CD45, CD3, CD19, CD20, CD56, CD16, HLA-DR, CD33, CD11b, CD14 and CD15, and BD Trucount beads for quantitation. Total MDSC were defined as CD45 + CD3^−^CD19^−^CD20^−^CD56^−^CD16^−^HLA-DR^−^CD33 + CD11b + cells, while the monocytic (M-MDSC) and polymorphonuclear subsets were defined as CD14+ or CD15+, respectively.

**Results:**

A novel gating strategy was devised to eliminate granulocytes and improve consistency in gating. Several pre-analytical variables were found to significantly affect MDSC quantitation, including collection tube type and time elapsed between blood collection and testing. Total and M-MDSC levels were a mean of 63% and 73% greater, respectively, with K_2_EDTA compared to Na^+^heparin collection tubes (*N* = 5). In addition, time elapsed at room temperature prior to cell labeling affected MDSC quantitation; by 24 h after blood collection, total and M-MDSC levels were a mean of 26% and 57% lower compared to testing as soon as possible after collection (*N* = 6). Refrigeration of samples at 4 °C ameliorated time-dependent effects at both 4 and 8 h, but not 24 h after blood collection. To establish normal ranges for this assay, MDSC levels were quantified in 67 healthy subjects (30 male, 37 female) ages 20–93. No significant differences in total or M-MDSC levels were detected for ages ≤60 compared to > 60 (*p* = 0.5 and *p* = 0.8, respectively). Finally, assay results demonstrated significantly higher MDSC levels among patients with hepatocellular carcinoma (*N* = 55) compared to age-matched healthy controls (*N* = 27) for total and M-MDSC (*p* = 0.006 and 0.004, respectively).

**Conclusions:**

MDSC are a heterogenous group of cells, and their quantitation in WB can be affected by a number of pre-analytical variables. Consideration of these factors, and measurement using a material type that has not been manipulated, such as whole blood, is likely to yield the most accurate results.

**Electronic supplementary material:**

The online version of this article (10.1186/s40425-019-0674-1) contains supplementary material, which is available to authorized users.

## Introduction

Myeloid-derived suppressor cells (MDSC) are a heterogeneous group of immature myeloid cells with profound immunosuppressive properties. They are thought to play an important role in normal homeostasis by limiting inflammation, but increased circulating levels of MDSC have also been associated with a panoply of disease states including cancer.

The expansion of MDSCs in nearly every type of cancer, and association with progressive disease, reduced survival, and poorer response to immunotherapy in melanoma and other cancer types [1–6] has sparked intense interest in this group of cells as both a biomarker and a therapeutic target. Unfortunately, the heterogeneity of the population, the lack of a single MDSC-specific surface marker, and the lack of consensus on the best combination of surface markers to define MDSCs by flow cytometry [7, 8], has hampered progress on its use as a biomarker.

A recent effort by the Association for Cancer Immunotherapy (CIMT) Immunoguiding Program described high variability between different laboratories for identifying and quantifying MDSC, with gating strategy being the main flow cytometric parameter associated with the variability [9]. We describe here a whole blood assay and a novel gating strategy that should decrease the ambiguity in identifying MDSC. Moreover, our characterization of the specimen collection and handling constraints for obtaining optimal results should provide guidance for best practices, potentially leading to decreased variability of results among different laboratories and studies.

## Materials and methods

### Human subjects

Peripheral blood from healthy and diseased subjects were obtained through a University of Washington Institutional Review Board (IRB)-approved protocol, #51834. Healthy enrolled subjects were self-identified, and denied use of immune-modulating medications, presence of immune conditions or recent infection. All enrolled subjects provided their written, voluntary informed consent. Additional samples tested were remnants of blood drawn in K_2_EDTA tubes, as permitted by the IRB-approved protocol. Healthy status, as described above, was determined by chart review. Diseased subjects were randomly selected and had hepatocellular carcinoma (HCC) with chronic hepatitis C virus (HCV) infection as the most common underlying disorder, and far fewer with hepatitis B virus (HBV), HCV/HBV coinfection, alcoholic hepatitis, or non-alcoholic steatohepatitis (NASH).

### Specimen collection and handling

Specimens were collected in various tube types and maintained at room temperature or at 4 °C until testing. Aliquots of fresh whole blood were utilized for real-time antibody labeling and flow cytometric analyses for the Whole Blood (WB) MDSC assay. PBMC were obtained using Lymphoprep™ media and SepMate™ isolation tubes (STEMCELL Technologies, Cambridge, MA).

### Flow cytometry

The WB MDSC 9-color, 11-parameter flow cytometric assay included the following fluorescently-labeled antibodies: CD45-V500-C (2D1), CD19-FITC (HIB19), CD20-FITC (L27), CD56-PECY7 (NCAM 16.2), HLA-DR-APC-H7 (L243), CD33-PE (WM53), CD11b-APC (ICRF44), and CD14-BV421 (MΦP9) (all, BD Biosciences); CD3-FITC (SK7), CD16-BV785 (3G8), and CD15-BV650 (W6D3) (all Biolegend). Samples were treated with BD FACS™ Lysing Solution (BD Biosciences) to lyse RBC. Total MDSC were defined as CD45 + CD3^−^CD19^−^CD20^−^CD56^−^CD16^−^HLA-DR^−^CD33 + CD11b + cells, while the monocytic (M-MDSC) and granulocytic or polymorphonuclear (PMN-MDSC) subsets were defined as CD14+ and CD15+, respectively. Absolute cell numbers were obtained using Trucount tubes (BD Biosciences). Presence of intracellular Ki67 was analyzed using antibodies to CD14 (MΦP9, exclusion marker), CD56 (NCAM16.2), CD4 (SK3), CD8 (SK1) (all, BD Biosciences); CD3 (SK7) and Ki67 (both, Biolegend) as well as Fixable Viability Dye eFluor 780 and FoxP3/Transcription Factor Staining Buffer Set (both eBioscience, San Diego, CA). All samples were fixed with 2% paraformaldehyde and data acquired on the same day. Flow cytometric data analysis was performed using a BD LSRFortessa and FlowJo software v9.9.5 (Treestar, Ashland, OR). Statistical analyses utilized paired or unpaired t tests, as appropriate.

### Suppression experiments

MDSC (“suppressors”) were enriched using negative selection for HLA-DR (anti-HLA-DR microbeads and LD columns, Miltenyi Biotec, Auburn, CA) and positive selection for CD33 (anti-CD33 microbeads and LS columns, Miltenyi Biotec) from a healthy donor’s fresh PBMC. HLA-DR^−^CD33^−^ cells were further depleted of CD3+ cells (anti-CD3 microbeads and LS columns, Miltenyi Biotec) and used as “non-suppressor controls”. An aliquot of autologous PBMC was set aside for use as the “responder” population. “Responders” were mixed with “suppressors” or “non-suppressor controls” and stimulated with anti-CD3/CD28 beads (1:1, Dynabeads,Thermo Fisher Scientific, Waltham, MA) for 4 days at 37 °C. CD3+ responder proliferation was measured using intracellular Ki67 labeling as described above.

## Results

### MDSC whole blood (WB) assay and gating strategy

**Fig. 1:**
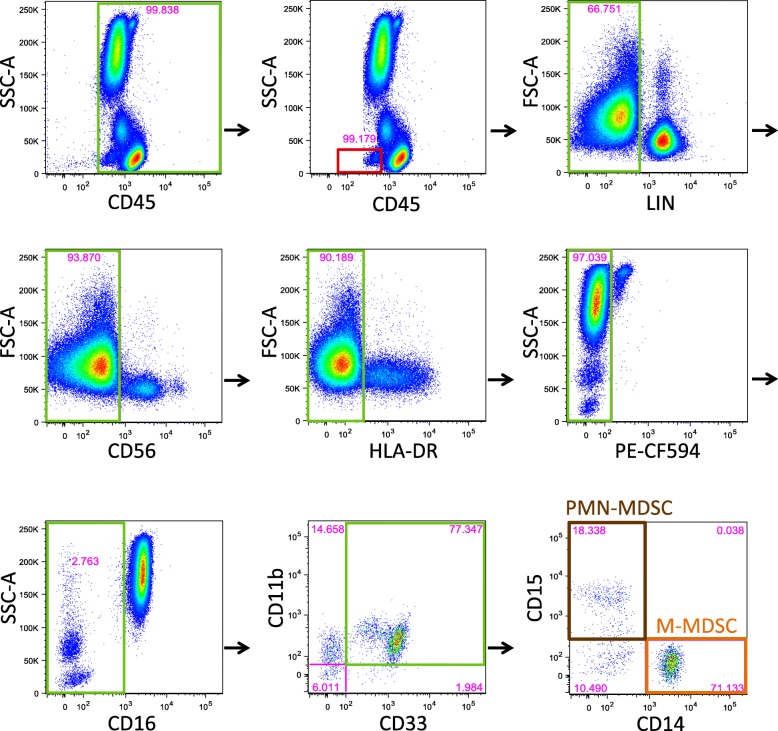
Gating strategy for identification of MDSC. Fresh whole blood (WB) samples (100 μL) served as the substrate for the WB flow cytometric assay. Green boxes indicate the cell populations that were selected for continued analysis. Red boxes indicate cell populations that were excluded. Initial exclusion of cellular doublets and debris, by gating on the singlets, is not shown. CD45+ cells were selected, followed by basophil exclusion, both using plots of CD45 vs SSC-A. Subsequently, T and B cells were excluded by gating on cells negative for pooled anti-CD3, anti-CD19 and anti-CD20 antibodies (lineage negative, LIN^−^) cells. NK cells were excluded by gating on CD56^−^ cells, and HLA-DR^−^ cells were selected. Eosinophils were excluded by gating on the PE-CF594^−^ cell population. Neutrophils were excluded by gating on CD16^−^ cells. Total myeloid derived suppressor cells (MDSC) were defined as CD33 + CD11b + cells. Polymorphonuclear-MDSCs (PMN-MDSC, a subset of total MDSCs, brown box) were identified by CD15+ expression, while monocytic-MDSCs (M-MDSC, a subset of total MDSCs, orange box) were identified by CD14+ expression. Early stage MDSC (e-MDSC), or counterpart MDSC in healthy individuals [7] are shown in the final plot, bottom left quadrant, as CD3/19/20/56^−^HLA-DR^−^CD16^−^CD33 + CD11b + CD14^−^CD15^−^ cells

We developed a 9-color, 11-parameter flow cytometric assay using whole blood as substrate. Our gating strategy began with the elimination of doublets and dead cells using forward (FSC) and side scatter (SSC-A) (Fig. 1). We then selected CD45+ cells using SSC-A on the y-axis. Basophils were excluded using their typical location on the CD45 vs SSC-A plot. To optimize the separation between negative and positive lineage populations, labeling with anti-CD56 antibody was analyzed separately from labeling with anti-CD3, anti-CD-19 and anti-CD-20 antibodies pooled together in the same fluorescence channel. HLA-DR-positive cells were excluded using a tight gate on the HLA-DR-negative population on an HLA-DR vs FSC plot. Compared to an HLA-DR vs CD14 plot, use of the FSC plot allowed for a “straighter edge” along which to consistently place the HLA-DR positive/negative threshold, leading to a slightly more stringent definition of MDSC. Also, it is worth noting that the use of an FMO plot to set the HLA-DR positive/negative threshold would have excluded cells that clearly fell within the HLA-DR-negative cluster of cells. We also took advantage of the observation that eosinophils exhibited autofluorescence in the PE-CF594 (PE-Texas Red) channel, so that these cells could be excluded without antibody labeling. CD16+ neutrophils were excluded by gating tightly against the major CD16+ population. Finally, total MDSCs were identified by co-expression of CD33 and CD11b, while polymorphonuclear (PMN-MDSC) and monocytic (M-MDSC) subpopulations were determined by CD15 and CD14 expression, respectively. Cells that did not express either CD14 or CD15 were considered MDSC counterparts (from healthy controls, as shown in Fig. 1) and are referred to as early stage-MDSC (e-MDSC) in disease patients [7].

**Fig. 2:**
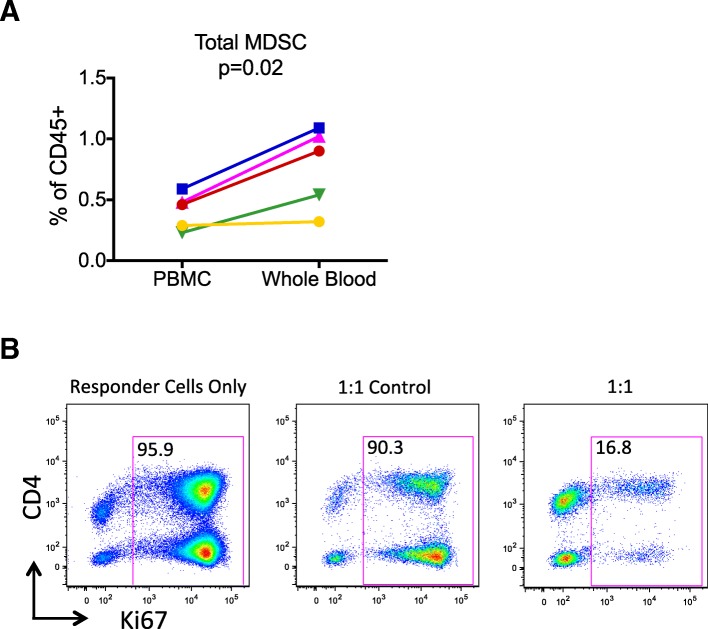
MDSC numbers in whole blood compared to PBMC, and immunosuppressive function. Total MDSC were measured using the same antibody panel in parallel using WB and PBMC from the same subjects, 2 healthy and 3 with HCC (**a**). Data are shown as the total MDSC percentage among CD45+ cells after imputing the number of CD45+ granulocytes to make the PBMC results comparable to the WB results. A paired t test was used to obtain the *p* value shown. To demonstrate suppressive activity of the MDSC identified by our assay, total MDSC (“suppressors”) were enriched as described in Materials and Methods, and cultured with autologous PBMC (“responders”) at a 1:1 ratio and stimulated with anti-CD3 and anti-CD28 beads for 4 days (right panel) (**b**). In addition, CD33^−^HLA-DR^−^CD3^−^ cells were used as control non-suppressor cells (middle panel). CD3+ T cell proliferation was detected using intracellular Ki67 labeling for all conditions, including “responder cells only” shown in the left panel

Since the vast majority of the human MDSC literature has utilized PBMC as the starting material, and associations with disease burden, survival, and other outcomes have been based upon results from PBMC, we sought to determine how MDSC quantification by our WB assay differed from using PBMC as the source material. In 5 unique samples, we compared total CD33 + CD11b + MDSC levels in WB and freshly isolated PBMC from the same donor, tested on the same day under the same conditions (Fig. 2A). Since density gradient isolation of PBMC results in the exclusion and pelleting of granulocytes, we imputed the total number of CD45+ cells for the MDSC frequency calculation (among PBMC) by using the ratio of lymphocytes to CD45+ cells obtained with the WB assay in whole blood. On average, the percentage of total MDSC among CD45+ cells was 1.9-fold greater using WB compared to PBMC (range 1.1–2.3, *p* = 0.02) as source material.

To confirm that the MDSC identified by our WB assay had immunosuppressive capability, PBMC were subjected to negative selection using HLA-DR-conjugated magnetic beads and subsequent positive selection using CD33-magnetic beads, leading to a 140-fold enrichment for total MDSC by flow cytometric analysis. When co-cultured with anti-CD3 and anti-CD28 bead-stimulated “responder” cells, the MDSC-enriched cells were able to suppress CD4+ and CD8+ T cell proliferation, as shown by intracellular Ki67 expression (Fig. 2B, far right panel). CD33-negative cells, obtained from the flow-through of the CD33 positive-selection column, were used as “control non-suppressor” cells (Fig. 2B, middle panel). This experiment was repeated 3 times with similar results.

### Preanalytical variables affect MDSC quantitation

In our analysis of preanalytical variables, we focused on total CD11b + CD33+ MDSC and M-MDSC because evaluation of both healthy individuals and hepatocellular carcinoma (HCC) patients demonstrated that the vast majority of MDSC were of the monocytic subtype, and very few were of the polymorphonuclear subtype. Therefore, very small changes such as 1 cell/μL could substantially affect the enumeration of PMN-MDSC from the WB assay.

**Fig. 3:**
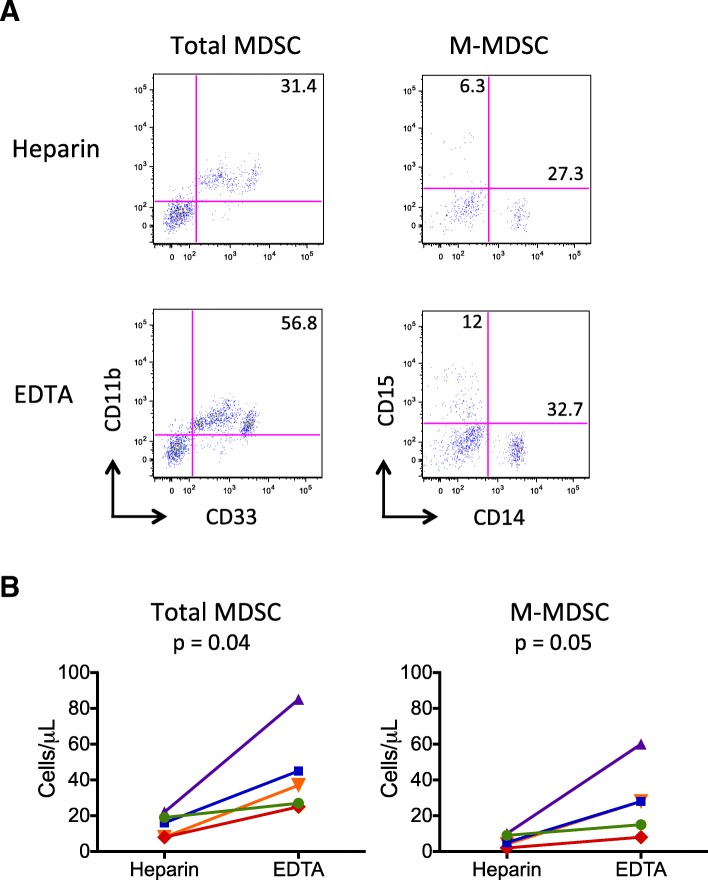
Collection tube type affects MDSC quantitation. Blood samples were simultaneously collected in Na^+^ heparin and K_2_EDTA tubes and tested using the WB assay. Representative plots of total and M-MDSC populations for samples collected from the same subject in the two tube types are shown (**a**). Quantitative results for the total and M-MDSC populations (cells/μL) from 5 unique individuals, 2 healthy and 3 with HCC, are shown (**b**). Mean percent differences between K_2_EDTA and Na^+^ heparin tubes for total MDSC and M-MDSC cell counts were 63% and 73%, respectively. *P*-values were obtained using paired t tests

**Fig. 4:**
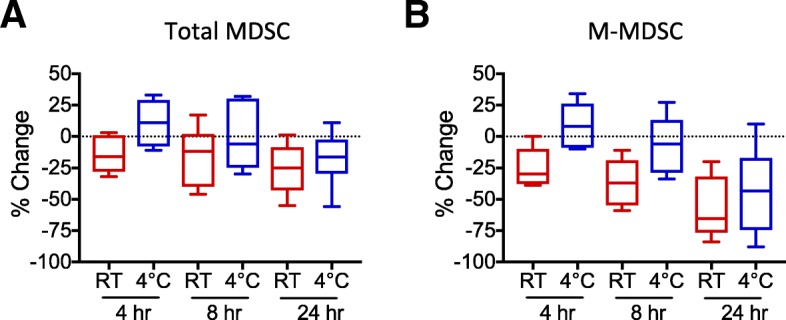
Temperature and time elapsed prior to testing affect MDSC quantitation. Whole blood samples from 2 healthy and 5 HCC subjects were collected in K_2_EDTA tubes and kept at room temperature (red box plots) or at 4 °C (blue box plots) before testing. Antibody labeling was conducted as soon as possible after blood collection, and % change in absolute numbers of total MDSC (**a**) and M-MDSC (**b**) were calculated between these baseline data and those obtained 4, 8, or 24 h after blood collection. Paired t tests were used to determine whether differences were statistically significant. At 4 h compared to baseline, mean percent change in MDSC levels for samples maintained at 4 °C versus room temperature (RT) were 9% vs − 15% change (*p* = 0.02) for total MDSC and 8% vs − 24% change (*p* = 0.009) for M-MDSC. At 8 h, mean percent changes for the 4 °C and RT samples were − 2% vs − 16% change (*p* = 0.06) for total MDSC and − 5% vs − 36% change (*p* = 0.006) for M-MDSC. Mean differences between the two conditions by 24 h were − 17% vs − 26% change (*p* = 0.3) for total MDSC and − 44% vs − 57% change (*p* = 0.4) for M-MDSC. Percent changes in MDSC counts by 24 h were lower than at 4 h (total MDSC *p* = 0.04 and M-MDSC *p* = 0.01) for samples maintained at 4 °C

Quantitation of total and M-MDSC was consistently higher in K_2_EDTA compared to heparin tubes (mean 63% and 73% greater, respectively) among 5 healthy and diseased donors with simultaneous blood collection in the two tube types, tested within 4 h of blood draw (Fig. 3B). The results obtained from K_2_EDTA versus heparin tubes were significantly different for both total MDSC (*p* = 0.04) and M-MDSC (*p* = 0.05). A representative example of these results is shown in Fig. 3A. Interestingly, substantial decreases in the relative frequencies of granulocytes and monocytes, but not lymphocytes, were seen immediately with blood collected in heparinized tubes compared to K_2_EDTA tubes (Additional file 1: Figure S1A), and expression of key MDSC-identifying surface markers such as CD11b on granulocytes and CD11b and CD33 on monocytes appeared to be more variable in heparinized tubes (Additional file 1: Table S1). In addition, the duration of time that WB was kept at room temperature prior to cell labeling affected the numbers of MDSC identified. Whole blood was collected in K_2_EDTA tubes and kept at room temperature or at 4 °C before testing (Fig. 4). Antibody labeling was conducted as soon as possible after blood collection (baseline), and % change in absolute numbers of total MDSC and M-MDSC were calculated. At 4 h after blood collection compared to baseline for both total and M-MDSC, samples maintained at 4 °C were found to have slightly increased numbers of MDSC than those maintained at room temperature (RT) (total MDSC: 9% vs − 15% change (*p* = 0.02) and M-MDSC: 8% vs − 24% change (*p* = 0.009)). At 8 h, differences were found between the 4 °C and RT samples (total MDSC: − 2% vs − 16% change (*p* = 0.06) and M-MDSC: − 5% vs − 36% change (*p* = 0.006)), although the difference between the two temperature conditions was greater for M-MDSC. No significant differences were found between the two conditions by 24 h for either total or M-MDSC (total MDSC: − 17% vs − 26% change (*p* = 0.3) and M-MDSC: − 44% vs − 57% change (*p* = 0.4)). However, MDSC counts by 24 h were significantly lower than at 4 h (total MDSC *p* = 0.04 and M-MDSC *p* = 0.01), for samples maintained at 4 °C. By contrast, for room temperature samples, the percent change by 24 h was only significant for M-MDSC (*p* = 0.02) but not for total MDSC (p = 0.3). M-MDSC counts were affected more by the passage of time at room temperature compared to total MDSC counts (*p* = 0.03, 0.02, and 0.01 for 4, 8 and 24 h, respectively). By contrast, levels of the combination of T (CD3+) and B (CD19+ or CD20+) cells measured at same time demonstrated no significant changes at 4, 8 or 24 h after blood collection at either temperature. In addition, it is useful to consider the effects of time and storage temperature in context; our average inter-assay coefficient of variation was 2.4 and 3.2% for total and M-MDSC, respectively. Similar results regarding the effects of time and temperature were found for samples collected in heparin tubes (data not shown).

During our evaluation of WB samples, we also found that hyperbilirubinemia and visible lipemia could have adverse effects on flow cytometric analyses of MDSC, specifically, as well as other cells. In some, but not all cases of either lipemia or hyperbilirubinemia, the data were difficult or impossible to interpret. Total bilirubin levels as low as 1.6 mg/dL (upper limit of normal =1.3 mg/dL) were found to impair the accurate identification of MDSCs, although untoward effects did not consistently appear at a given total bilirubin level (data not shown).

### Lack of effect of subject age on MDSC levels among healthy controls

**Fig. 5:**
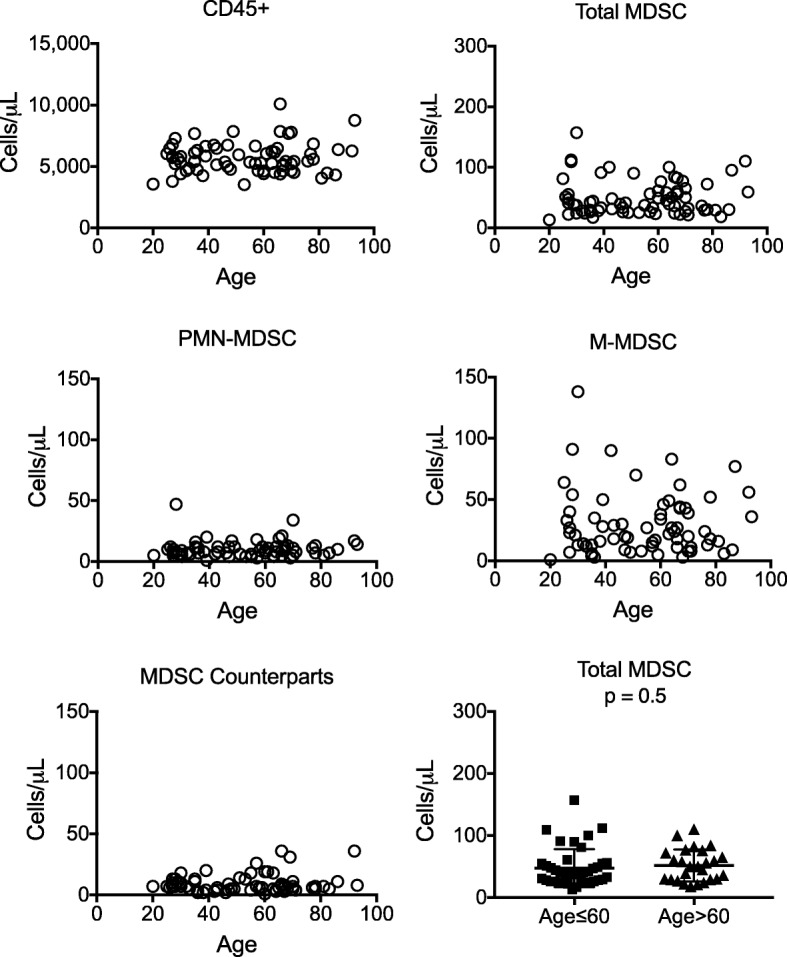
MDSC frequencies among healthy adults. Blood samples were collected from 67 healthy subjects (30 males, 37 females) ages 20 to 93 in K_2_EDTA tubes. Quantitative results for CD45+, CD33 + CD11b + (total MDSC), M-MDSC (CD14+), PMN-MDSC (CD15+), MDSC counterparts (CD14^−^CD15^−^) from each subject are shown as individual symbols. No significant difference was found in total MDSC levels among subjects (both genders) 60 years and younger (*N* = 41) compared to those over the age of 60 (*N* = 26) (unpaired t test, *p* = 0.5, bottom right panel)

It was important to determine a normal range for MDSCs among our local healthy population across a wide range of ages and for both genders (Fig. 5). Samples from 67 healthy subjects (30 male, 37 female) ages 20–93 were tested using the WB assay, after accounting for the preanalytical variables described above. Contrary to expectation, no significant differences in total or M-MDSC levels were detected with age ≤ 60 compared to > 60 (*p* = 0.5 and *p* = 0.8, respectively). Normal thresholds were set to include 95% of results from healthy volunteers, as is standard for clinical assays.

### A subset of hepatocellular carcinoma (HCC) patients harbor higher MDSC levels compared to age-matched control subjects

**Fig. 6:**
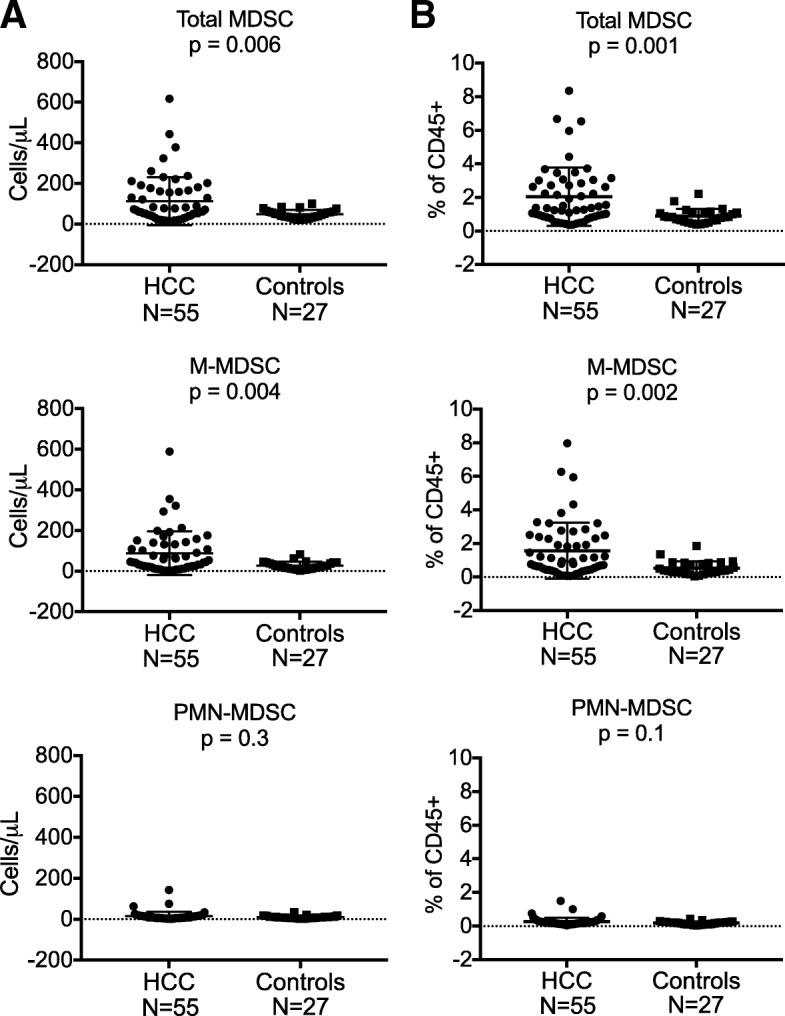
MDSC frequencies are higher among hepatocellular carcinoma (HCC) patients compared to age-matched control subjects. Whole blood samples collected from HCC patients (*N* = 55, mean age = 62.6, range 50–75) and healthy controls (*N* = 27, mean age 63.2, range 48–71) were tested using the WB MDSC assay. Results for total MDSC (CD11b + CD33+, p = 0.006) and M-MDSC (CD11b + CD33 + CD14 + CD15^−^, *p* = 0.004) counts (cells/μL) among HCC patients were significantly different from healthy controls, but not for PMN-MDSC (CD11b + CD33 + CD14^−^CD15+, *p* = 0.3, **a**). Corresponding data for total MDSC, M-MDSC, and PMN-MDSC displayed as % of CD45+ cells is also shown (**b**). Using absolute cell counts, twenty of the 55 HCC patients (37%) had total MDSC levels above our normal threshold of 110 cells/μL, while 19 (35%) and 5 (9%) had M-MDSC and PMN-MDSC levels above our normal thresholds of 90 cells/μL and 25 cells/μL, respectively. All *p*-values were obtained using unpaired t tests

To confirm that our WB assay detects differences in MDSC levels between cancer patients and healthy controls, whole blood samples from HCC patients (*N* = 55, mean age = 62.6, range 50–75) and age-matched healthy controls (*N* = 27, mean age 63.2, range 48–71) were obtained (Fig. 6). Results for total MDSC (CD11b + CD33+) and M-MDSC (CD11b + CD33 + CD14+) counts (cells/μL) were significantly different between the two groups (*p* = 0.006 and *p* = 0.004, respectively) but not for PMN-MDSC (*p* = 0.3). Similar differences were found when the same data were considered as % of CD45+ cells. Of the 55 HCC patients, 51 had been diagnosed with cirrhosis. The underlying causes of liver disease were identified as: 45 with current or past HCV infection, 2 with HBV infection, 1 with HCV and HBV coinfection, 6 with non-alcoholic steatohepatitis (NASH), and 2 with chronic alcohol intake. Twenty of the 55 HCC patients (37%) had total MDSC levels above our normal threshold of 110 cells/μL while 19 (35%) and 5 (9%) had M-MDSC and PMN-MDSC levels above our normal thresholds of 90 and 25 cells/μL, respectively. Elevated total and M-MDSC levels were highly correlated; only 1 subject had an elevated total MDSC level of 161, where the M-MDSC level of 76 did not surpass the normal threshold for M-MDSC, but the PMN-MDSC level was high at 63. M-MDSC comprised a mean of 81% of total MDSC among 20 patients with elevated levels of total MDSC. In no case were M-MDSC or PMN-MDSC levels elevated and the total MDSC level within the normal range.

## Discussion

The WB MDSC assay described here allows for a straightforward and robust analysis strategy to exclude irrelevant cell populations including neutrophils, basophils, and eosinophils, and identify clear thresholds between positive and negative cell populations. Moreover, this assay identifies a greater number of MDSC in WB compared to PBMCs. Careful evaluation of assay performance also reveals a number of pre-analytical factors that can significantly affect the quantitation of MDSC; collection tube type, time elapsed between venipuncture and antibody labeling and the temperature at which samples are maintained until antibody labeling were all found to be important variables to control in order to obtain accurate and reproducible results. Thresholds for MDSC levels among healthy individuals were identified, and no substantial increase in MDSC frequencies were found with advanced age. As expected, higher MDSC levels were identified in a subset of patients with hepatocellular carcinoma, compared to healthy controls of similar age.

While all investigators may not agree on the surface markers that should be used to identify the heterogeneous MDSC population, our panel incorporates the most commonly utilized surface markers from published reports of MDSC [6, 8] and meets the minimal phenotypic characteristics necessary to identify MDSC proposed by Gabrilovich and colleagues [7]. Due to our use of whole blood as the starting material rather than PBMC, we were obliged to exclude polymorphonuclear leukocyte populations analytically, rather than through density gradient centrifugation. Systematic exclusion of basophils, eosinophils and CD16 + SSC^pos-hi^ neutrophils decreases the total MDSC numbers, and substantially decreases the CD14^−^CD15^−^ MDSC (e-MDSC) subpopulation. We recognize that our gating strategy leans toward a tighter definition of MDSC, particularly with regard to PMN-MDSC. Although we did not find elevated levels of PMN-MDSC in liver cancer patients, we have in patients treated with immunosuppressive medications (data not shown). We acknowledge that our definition of PMN-MDSC may differ from that of others. Specific markers of PMN-MDSC in whole blood have yet to be established, but LOX-1 appears to be a promising marker that has been found on a subset of immunosuppressive PMN-MDSC [10] and is worthy of future investigation. Finally, our data confirm the immunosuppressive function of the cells we identified as MDSC, and our results in patients with liver cancer compared to healthy controls are similar to those that have been published. It is worth noting that these other groups also previously demonstrated no differences in MDSC levels among healthy, HCV-infected non-cirrhotic, and HCV-infected cirrhotic subjects [11, 12]. Together, our results provide evidence that the WB assay identifies the immunosuppressive MDSCs that many other groups have studied previously.

An important variable in obtaining reproducible flow cytometric results is the analytical step of gating. Particularly true for complex panels that require multiple subsetting steps, small changes in how positive and negative populations are defined can have a substantial impact on results. Thus, utilizing antibody clones and fluorochromes to optimize the separation of positive and negative populations is an important factor in assay design, keeping specific flow cytometer configurations in mind. In addition, a key surface marker necessary for defining MDSC populations is HLA-DR; unfortunately, HLA-DR surface expression often appears as a continuous spectrum, making the identification of a positive/negative threshold difficult. An important advance afforded by our panel design and gating strategy was improved clarity and consistency in identifying HLA-DR-negative cells.

We developed this WB assay with the goal of offering it as a clinical test, rather than as a research assay. Dispensing with the need for density gradient centrifugation results in a significant decrease in the time required to perform the testing in real-time, decreases the source material needed to conduct the testing, and decreases the amount of manipulation of the starting material that likely affects MDSC quantitation. Finally, direct quantitation of whole blood MDSC levels is more likely to be comparable between different laboratories [13].

In the course of characterizing assay performance, we identified a number of factors that adversely affect MDSC quantitation by our assay. Visible lipemia and high total bilirubin levels appeared to complicate data analyses in some cases, which was not surprising, given the known refractive effects of lipemia [14] and the ability of bilirubin to induce autofluorescence [15]. A major issue for MDSC quantitation was the effect of time elapsed between specimen collection and assay initiation (antibody labeling). In a clinical laboratory setting within a large multi-site medical system, it was impractical to expect that a sample could be consistently delivered to the laboratory and testing initiated within less than 4 h. Due to this practicality and supported by data from others [16], we set 4 h as our gold standard. Others have described alterations in flow cytometric measurement of myeloid cells over days [17], but we were surprised to discover that even by 8 h after blood collection, a decrement in MDSC numbers was consistently detected. We speculate that changes in the expression patterns of labile surface molecules used to define MDSC such as HLA-DR [18] and CD11b [19] and/or cell death, likely contributed to both increases and decreases in MDSC numbers detected over time. Storing the samples upon receipt in the laboratory at 4 °C appeared to ameliorate the effect of storage time at room temperature, which we have now incorporated into our regular practice. Moreover, many researchers collect whole blood in heparinized green top tubes, which we found to be inferior to K_2_EDTA tubes for maintaining MDSC levels over time. Implementation of processes to offset these deleterious factors deviates from current standard practices used for handling whole blood obtained from patients participating in many local and multi-center clinical trials; in these settings whole blood is often maintained at room temperature and processed or tested within but often close to 24 h after collection. Our data argue that if MDSC quantitation is an important biomarker of studies, then optimization of its measurement will require a commitment to process changes. Based upon the data shown here, we believe that whole blood kept at room temperature for ≥24 h is unlikely to yield accurate MDSC frequencies. Similar to best practices for PBMC processing used by the global laboratories of the HIV Vaccine Trials Network (HVTN) [20], we suggest that local laboratories should develop the expertise to conduct this assay, so that deleterious effects of pre-analytical factors can be lessened. Moreover, external quality assurance testing will ensure comparability of results among laboratories.

Although multiple studies have clearly identified increases in MDSC levels in blood, bone marrow, and secondary lymphoid organs with aged compared to young mice [21–23], very little data has been available in humans. In the single study that has been cited by several sources, Verschoor et al. reported that HLA-DR^−^CD33+ MDSC frequencies among cryopreserved PBMC were elevated in 45 seniors (ages 61–76) compared to 41 younger individuals (ages 19–59) [24]. While the difference between the groups was statistically significant (*p* < 0.05), the values shown mostly overlapped. We analyzed fewer subjects (41 subjects ages 20–60 and 26 subjects ages > 60–93) but did not find any statistically significant difference in MDSC levels between subjects ≤60 or > 60 (*p* = 0.5). Differences in the source material (whole blood vs cryopreserved PBMC), definition of MDSC (HLA-DR^−^CD33+ vs CD3/19/20/56^−^HLA-DR^−^CD16^−^CD33 + CD11b + cells), and the criteria for health in the two healthy cohorts could have contributed to the different results obtained. Additional future studies will be necessary to further evaluate this issue.

Our data raise questions about existing MDSC data that have been reported in the literature. For many research laboratories, conducting MDSC testing in real time, within 8 h of venipuncture, may not be a practical option. Based on previous reports from multiple groups corroborating associations between MDSC levels and outcome (despite using different surface markers to identify MDSC) [6] and encouraging comparative data from fresh and frozen samples [25], we are optimistic that MDSC levels detected using older blood (> 24 h since blood collection) and/or cryopreserved PBMC, when collected in a consistent manner, can produce reproducible results. However, it is possible that weaker associations with MDSC levels that are clinically important may not be discovered as a result of the limitations of how testing was performed.

We conclude that accurate and reproducible measurement of MDSC levels can be challenging, and is related to variation in cell surface marker expression that occurs with specimen handling and with time elapsed ex vivo prior to testing. However, given the central role that MDSC have been shown to play in mediating control of immune responses, and the potential importance of this cell type as a biomarker and/or target in a large number of disease states, efforts to optimize and harmonize its measurement across many labs will be important. The whole blood assay described here yields a quantitative result, minimizes specimen processing thus decreasing the cost of testing, reduces the specimen volume requirement to a negligible 100 μL, and utilizes a robust and consistent gating strategy. Future studies that include optimized measurement of MDSC levels as described here, in conjunction with collection of clinical outcome measures, are needed to refine the clinical utility of this testing.

## Conclusions

Characterization of our novel whole blood MDSC assay revealed the profound effect of pre-analytical factors on obtaining optimal results from this heterogeneous and fluid cell population. These factors included minimal specimen handling, blood collection in K_2_EDTA tubes, sample storage at 4 °C, and testing within 8 h of collection. This information should aid efforts to improve and harmonize flow cytometric analyses of this important cell type.

## Additional file

Additional file 1: **Figure S1** Effects of tube type and time elapsed after blood collection on proportions of granulocytes, monocytes, and lymphocytes in whole blood. **Table S1** Percentage Differences in Cell Types and Surface Marker Expression Between Heparin and EDTA Tubes (*N* = 5). (PPTX 1550 kb)

## Data Availability

All data generated or analyzed during this study are included in this published article.
