# Supplementary material for: Characterization of a whole blood assay for quantifying myeloid-derived suppressor cells
Source: J Immunother Cancer. 2019 Aug 28;7:230. doi: 10.1186/s40425-019-0674-1 (PMC6714080; doi:10.1186/s40425-019-0674-1)
Supplement: Supplementary file 1 — Figure S1 Effects of tube type and time elapsed after blood collection on proportions of granulocytes, monocytes, and lymphocytes in whole blood. Table S1 Percentage Differences in Cell Types and Surface Marker Expression Between Heparin and EDTA Tubes (N = 5). (PPTX 1550 kb) [file 40425_2019_674_MOESM1_ESM.pptx]

## Slide 1
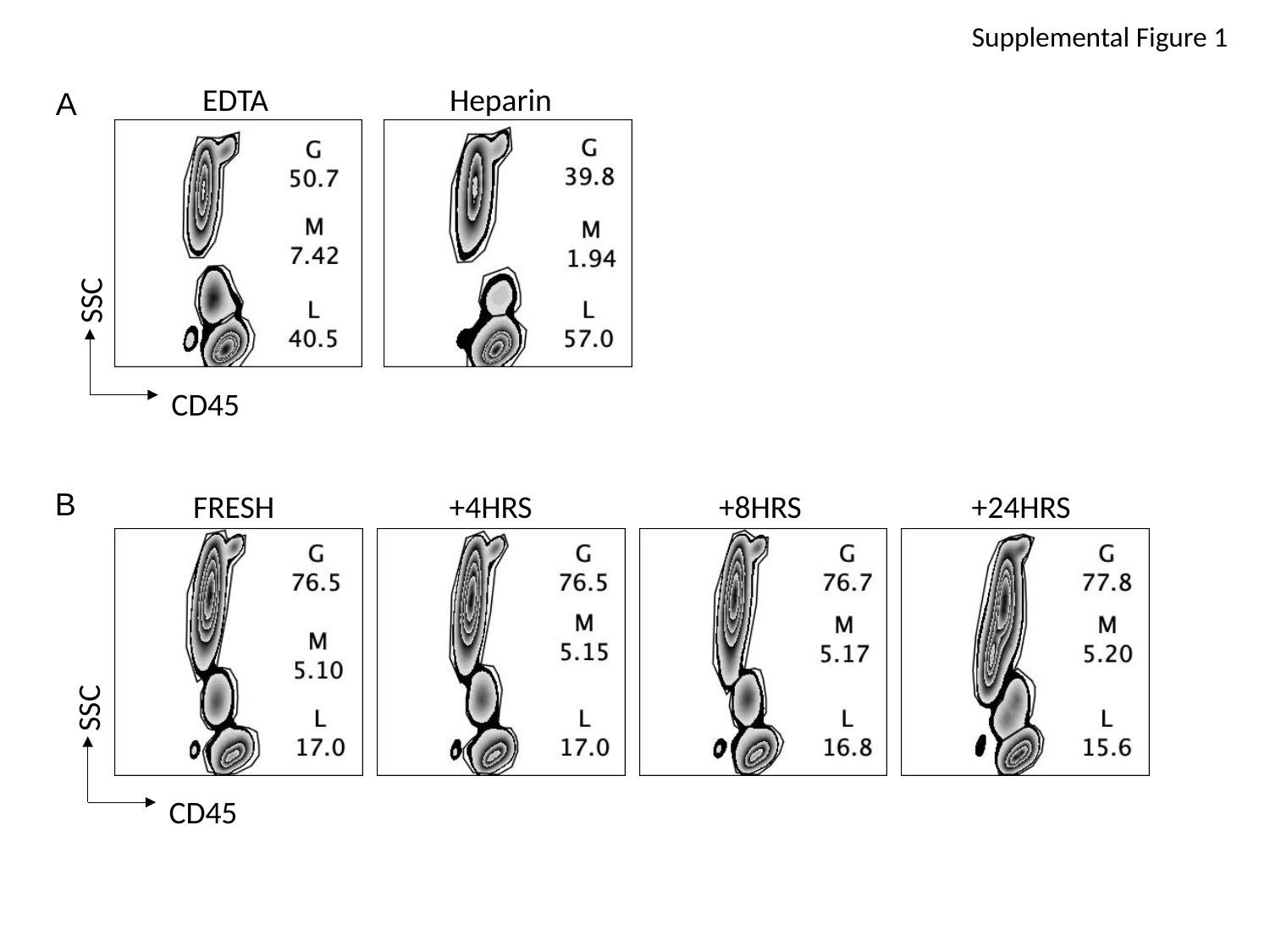

Supplemental Figure 1
EDTA
Heparin
A
B
SSC
CD45
FRESH	 +4HRS +8HRS	 +24HRS
SSC
CD45

## Slide 2
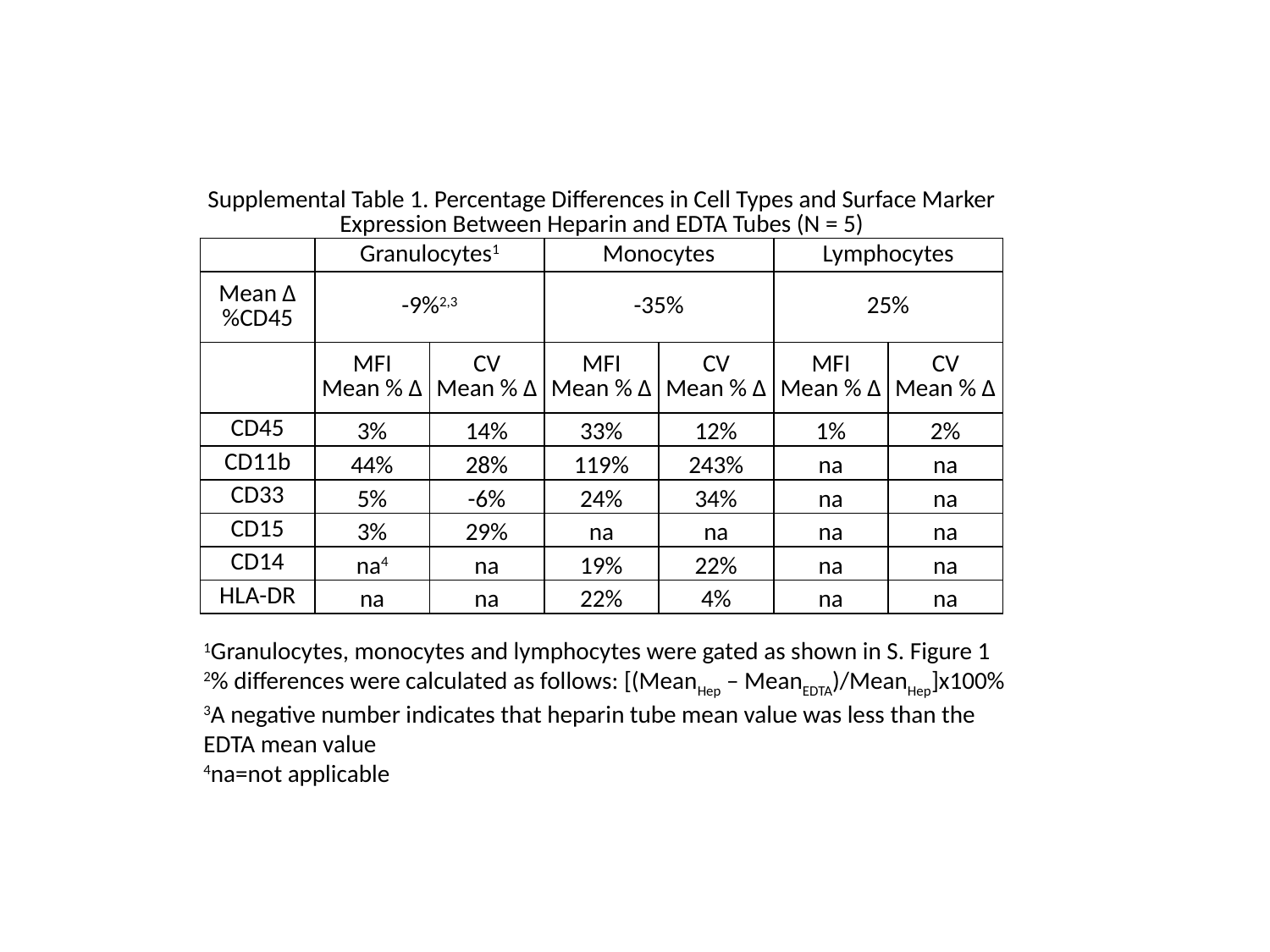

| Supplemental Table 1. Percentage Differences in Cell Types and Surface Marker Expression Between Heparin and EDTA Tubes (N = 5) | | | | | | |
| --- | --- | --- | --- | --- | --- | --- |
| | Granulocytes1 | | Monocytes | | Lymphocytes | |
| Mean ∆ %CD45 | -9%2,3 | | -35% | | 25% | |
| | MFI Mean % ∆ | CV Mean % ∆ | MFI Mean % ∆ | CV Mean % ∆ | MFI Mean % ∆ | CV Mean % ∆ |
| CD45 | 3% | 14% | 33% | 12% | 1% | 2% |
| CD11b | 44% | 28% | 119% | 243% | na | na |
| CD33 | 5% | -6% | 24% | 34% | na | na |
| CD15 | 3% | 29% | na | na | na | na |
| CD14 | na4 | na | 19% | 22% | na | na |
| HLA-DR | na | na | 22% | 4% | na | na |
1Granulocytes, monocytes and lymphocytes were gated as shown in S. Figure 1
2% differences were calculated as follows: [(MeanHep – MeanEDTA)/MeanHep]x100%
3A negative number indicates that heparin tube mean value was less than the EDTA mean value
4na=not applicable
